# Safety and effectiveness of taliglucerase alfa in patients with Gaucher disease: an interim analysis of real-world data from a multinational drug registry (TALIAS)

**DOI:** 10.1186/s13023-022-02289-7

**Published:** 2022-04-01

**Authors:** Lina Titievsky, Tilman Schuster, Ronnie Wang, Muhammad Younus, Andrew Palladino, Kabir Quazi, Michael P. Wajnrajch, Betina Hernandez, Pamela S. Becker, Neal J. Weinreb, Christina Chambers, Roy Mansfield, Louise Taylor, Li-Jung Tseng, Paige Kaplan

**Affiliations:** 1 Pfizer, Inc., New York, NY USA; 2 Pfizer Inc, 500 Arcola Road, Collegeville, PA 19426 USA; 3 New York University Grossman School of Medicine, New York, NY USA; 4 University of California, Irvine, Irvine, CA USA; 5 University of Washington School of Medicine, Seattle, WA USA; 6 University of Miami Miller School of Medicine, Miami, FL USA; 7 University of California, San Diego, La Jolla, CA USA; 8 The Children’s Hospital of Philadelphia, Philadelphia, PA USA

**Keywords:** Taliglucerase alfa, Gaucher disease, Effectiveness, Non-interventional study, Safety

## Abstract

**Background:**

Limited real-world data from routine clinical care are available on the safety and effectiveness of treatment with taliglucerase alfa in patients with Gaucher disease (GD).

**Methods:**

Taliglucerase Alfa Surveillance (TALIAS), a multinational prospective Drug Registry of patients with GD, was established to evaluate the long-term safety (primary objective) and effectiveness (secondary objective) of taliglucerase alfa. We present an interim analysis of the data from the Drug Registry collected over the 5-year period from September 2013 to January 2019.

**Results:**

A total of 106 patients with GD (15.1% children aged < 18 years; 53.8% females) treated with taliglucerase alfa have been enrolled in the Drug Registry, as of January 7, 2019. The median duration of follow-up was 795 days with quartiles (Q1, Q3) of 567 and 994 days. Fifty-three patients (50.0%) were from Israel, 28 (26.4%) were from the United States, and 25 (23.6%) were from Albania. At the time of enrollment, most patients (87.7%) had received prior enzyme replacement therapy (ERT). Thirty-nine of the 106 patients had treatment-emergent adverse events (AEs). Twelve of the 106 patients experienced serious AEs; two patients experienced four treatment-related serious AEs. Four patients died, although none of the deaths was considered to be related to taliglucerase alfa treatment by the treating physicians. Nine patients discontinued from the study, including the four who died. At baseline, patients with prior ERT had a higher mean hemoglobin concentration and platelet counts than treatment-naïve patients, likely reflecting the therapeutic effects of prior treatments. During follow-up, the hemoglobin concentration and platelet counts increased in the treatment-naïve patients and remained relatively constant or increased slightly in patients with prior ERT. Spleen and liver volumes decreased in treatment-naïve patients.

**Conclusions:**

The interim data showed no new or emergent safety signals. The overall interim data are consistent with the clinical program experience and known safety and effectiveness profile of taliglucerase alfa.

**Supplementary Information:**

The online version contains supplementary material available at 10.1186/s13023-022-02289-7.

## Background

Gaucher disease (GD) is a rare, inherited lysosomal storage disorder. Common manifestations of GD include hepatosplenomegaly, anemia, thrombocytopenia, skeletal pathology, and, less frequently, lung disease [1]. The National Organization for Rare Disorders estimates that approximately 6000 individuals have GD in the United States [2]. GD is the most common genetic disorder of persons with Ashkenazic Jewish ancestry; the incidence in this population may be as high as one in 450 births [2]. There are 3 subtypes of GD; Type 1 GD, the most common subtype, is non-neuronopathic, and Types 2 and 3 are neuronopathic [3].

Although GD does not yet have a cure, the available treatments help to control symptoms, prevent, or ameliorate organ damage and improve length and quality of life. The two types of pharmacotherapy currently used to treat patients with GD include enzyme replacement therapy (ERT), which supplements deficient mutant glucocerebrosidase by intravenous infusion of recombinant active enzyme, and oral substrate reduction therapy (SRT), which reduces the amount of stored glucocerebroside by inhibiting its biosynthesis [2, 4].

First approved in 2012 in the United States [5], taliglucerase alfa for injection is a recombinant active form of the human lysosomal enzyme acid β-glucosidase indicated for long-term ERT in adult and pediatric patients aged 4 to < 18 years with Type 1 GD in a number of countries, including Australia, Canada, and the United States [6], and in pediatric patients with Type 3 GD in several countries, including Canada, Colombia, Taiwan, and Ukraine [7], based on clinical trial data of patients aged 2 to < 18 years with GD [8, 9]. Clinical trials demonstrated that taliglucerase alfa is efficacious and generally well tolerated, with transient adverse events (AEs) ranging from mild to moderate severity [8–14]. The established safety profile of taliglucerase alfa is similar to other approved ERTs; however, the validity and generalizability of clinical trial data are limited by strict eligibility criteria, relatively short durations of follow-up, and small sample sizes. Therefore, post-approval data collection is critical for evaluating safety and effectiveness of taliglucerase alfa in routine clinical care and in various patient subgroups, including those with previous ERT or SRT, pediatric patients, or pregnant women.

Taliglucerase Alfa Surveillance (TALIAS), a multinational, prospective drug registry of patients with GD receiving taliglucerase alfa (hereafter referred to as Drug Registry), was established in 2013 as a post-marketing requirement to the US Food and Drug Administration [15]. Patients with GD Types 1, 2, or 3 were eligible for inclusion. The primary objective of this Drug Registry is to characterize the long-term safety profile of taliglucerase alfa through solicited collection of AEs; the secondary objective is to evaluate the effectiveness of taliglucerase alfa through assessments of hematological biomarkers and organ volumes. In addition, a pregnancy and lactation sub-study nested within the Drug Registry is being conducted among female patients to further characterize the potential impact of taliglucerase alfa exposure in utero on pregnancy outcomes and the newborn, and the potential impact of taliglucerase alfa exposure on infant outcomes through breast milk. This report presents results of a planned interim analysis of data from the Drug Registry collected over a 5-year period.

## Results

### Baseline patient demographics and clinical characteristics

A total of 106 patients (adults: *n* = 90, mean age: 49.7 years; pediatric: *n* = 16, mean age: 11.2 years) were enrolled in the Drug Registry as of January 7, 2019. Of the 106 patients, 102 (96.2%; pediatric patients, n = 13; adult patients, n = 89) were diagnosed with Type 1 GD, with eight treatment-naïve patients and 94 treatment-experienced patients, while four (3.8%; pediatric patients, n = 3; adult patients, n = 1) were diagnosed with Type 3. The four patients with Type 3 GD were treatment experienced. Fifty-three (50.0%) patients were from Israel, 28 (26.4%) were from the United States, and 25 (23.6%) were from Albania (Table 1). Investigational sites located in Turkey started enrolling patients after the data cutoff date for this interim analysis. Most patients (87.7%) had received prior ERT. Among the 90 adult patients, six were treatment naïve and 84 were treatment experienced (79 had prior ERT exclusively; five had prior ERT and SRT). Among the 16 pediatric patients, two were treatment naïve and 14 were treatment experienced (all 14 received prior ERT exclusively). The median duration of follow-up of patients was 795 days with quartiles (Q1, Q3) of 567 and 994 days. No pediatric patients discontinued the study. Nine (8.5%) patients, all adults, discontinued the study; reasons for study discontinuation included lost to follow-up (*n* = 5) and death (*n* = 4). Of the patients who died, all were in the prior ERT group; one had previously discontinued treatment due to throat irritation/throat tightness (59-year-old female), two patients (73-year-old female and 74-year-old male) had previously discontinued treatment due to patient preference, and one patient (72-year-old female) died on day 830 at the hospital due to abdominal disseminated carcinoma. Based on the weak temporal relationship and known safety profile of the study drug, the investigator considered the events to be unrelated to the study medication.

**Table 1:** Demographics and clinical characteristics of study patients

Patient characteristic		Drug Registry cohort (*N* = 106)
Sex, *n* (%)		
Male		49 (46.2)
Female		57 (53.8)
Age category (years)		
< 12		8 (7.5)
12–< 18		8 (7.5)
18–44		36 (34.0)
> 44–64		32 (30.2)
≥ 65		22 (20.8)
Mean age (years)		
< 18		11.2
≥ 18		49.7
Race, *n* (%)		
White		94 (88.7)
Black		1 (0.9)
Other		11 (10.4)
Ethnicity, *n* (%)		
Ashkenazi Jewish		61 (57.5)
Geographic location, *n* (%)		
Israel		53 (50.0)
United States		28 (26.4)
Albania		25 (23.6)
Employment status, *n* (%)		
Currently working (adult patients only, *n* = 90)		47 (52.2)
Gaucher disease diagnosis, *n* (%)		
Type 1		102 (96.2)
Type 2		0 (0.0)
Type 3		4 (3.8)
Symptoms at Gaucher disease diagnosis, *n* (%)		
Splenomegaly		86 (81.1)
Thrombocytopenia		63 (59.4)
Hepatomegaly		49 (46.2)
Anemia		45 (42.5)
Fatigue		39 (36.8)
Bone pain		36 (34.0)
Other symptoms		29 (27.4)
Comorbidities/conditions at enrollment, *n* (%)		
Surgical and medical procedures		31 (29.2)
Vascular disorders including hemorrhage		31 (29.2)
Musculoskeletal/connective tissue disorders, including osteoporosis		29 (27.4)
Cardiac disorders		25 (23.6)
Hepatobiliary disorders		12 (11.3)
Psychiatric disorders		10 (9.4)
Splenectomy at enrollment, *n* (%)		
Yes		14 (13.2)
Historical ERT, *n* (%)		
Taliglucerase alfa		94 (95.9)
Imiglucerase		57 (58.2)
Velaglucerase alfa		14 (14.3)
Alglucerase		3 (3.1)

*ERT* enzyme replacement therapy

### Safety assessment

#### SAEs

Serious AEs (SAEs) occurred in 12 adult patients (11.3%; Table 2). No SAEs were reported in children. Two of 12 patients experienced four SAEs that were considered related to taliglucerase alfa by the treating physician (i.e. systemic lupus erythematosus in a 36-year-old female who had Hashimoto’s thyroiditis; chest pain, back pain, and dyspnea in a male aged 34 years who experienced these symptoms after the second taliglucerase alfa infusion. Taliglucerase alfa was discontinued in these patients with consequent resolution of the SAEs. The other SAEs experienced by 10 patients were considered not related to taliglucerase alfa by the treating physicians.

**Table 2:** Summary of TEAEs and all-cause mortality by treatment group

	Treatment-naïve group (*n* = 8)	Prior ERT group (*n* = 93)	Prior ERT and SRT group (*n* = 5)^a^	Total patients (*N* = 106)
Total TEAEs, *n*	15	78	2	95
Total treatment-related TEAEs, *n*	1	20	0	21
Patients with TEAEs, *n* (%)	4 (50.0)	34 (36.6)	1 (20.0)	39 (36.8)
Patients with treatment-related TEAEs, *n* (%)	1 (12.5)	11 (11.8)	0 (0.0)	12 (11.3)
Patients with SAEs, *n* (%)	1 (12.5)	10 (10.8)	1 (20.0)	12 (11.3)
Patients with treatment-related SAEs, *n* (%)	0 (0.0)	2 (2.2)	0 (0.0)	2 (1.9)
Patients with severe TEAEs	1 (12.5)	6 (6.5)	1 (20.0)	8 (7.5)
Patients with treatment-related severe TEAEs	0 (0.0)	2 (2.2)	0 (0.0)	2 (1.9)
Patients with discontinuations due to TEAEs^b^, *n* (%)	0 (0.0)	4 (4.3)	0 (0.0)	4 (3.8)
Patients with all-cause mortality, *n* (%)	0 (0.0)	4 (4.3)	0 (0.0)	4 (3.8)

*ERT* enzyme replacement therapy; *SAEs* serious adverse events; *SRT* substrate reduction therapy; *TEAEs* treatment-emergent adverse events

^a^One patient was reported under the “Prior ERT and SRT” group due to erroneous recording of some ERTs as SRTs in the study database

^b^The number of patients who discontinued due to all causality TEAEs are same as the ones due to treatment-related TEAEs

For the four adult patients who died, three had discontinued taliglucerase alfa > 18 months before their death, and the fourth patient died 20 days after discontinuing taliglucerase alfa. The causes of death for these four patients were sepsis due to urinary tract infection, multiple organ dysfunction, unknown cause, and abdominal malignancy, all of which were considered unrelated to taliglucerase alfa by the treating physician. With respect to the unknown cause of death, the patient died > 3.5 years after discontinuing taliglucerase alfa treatment.

#### TEAEs

**Fig. 1:**
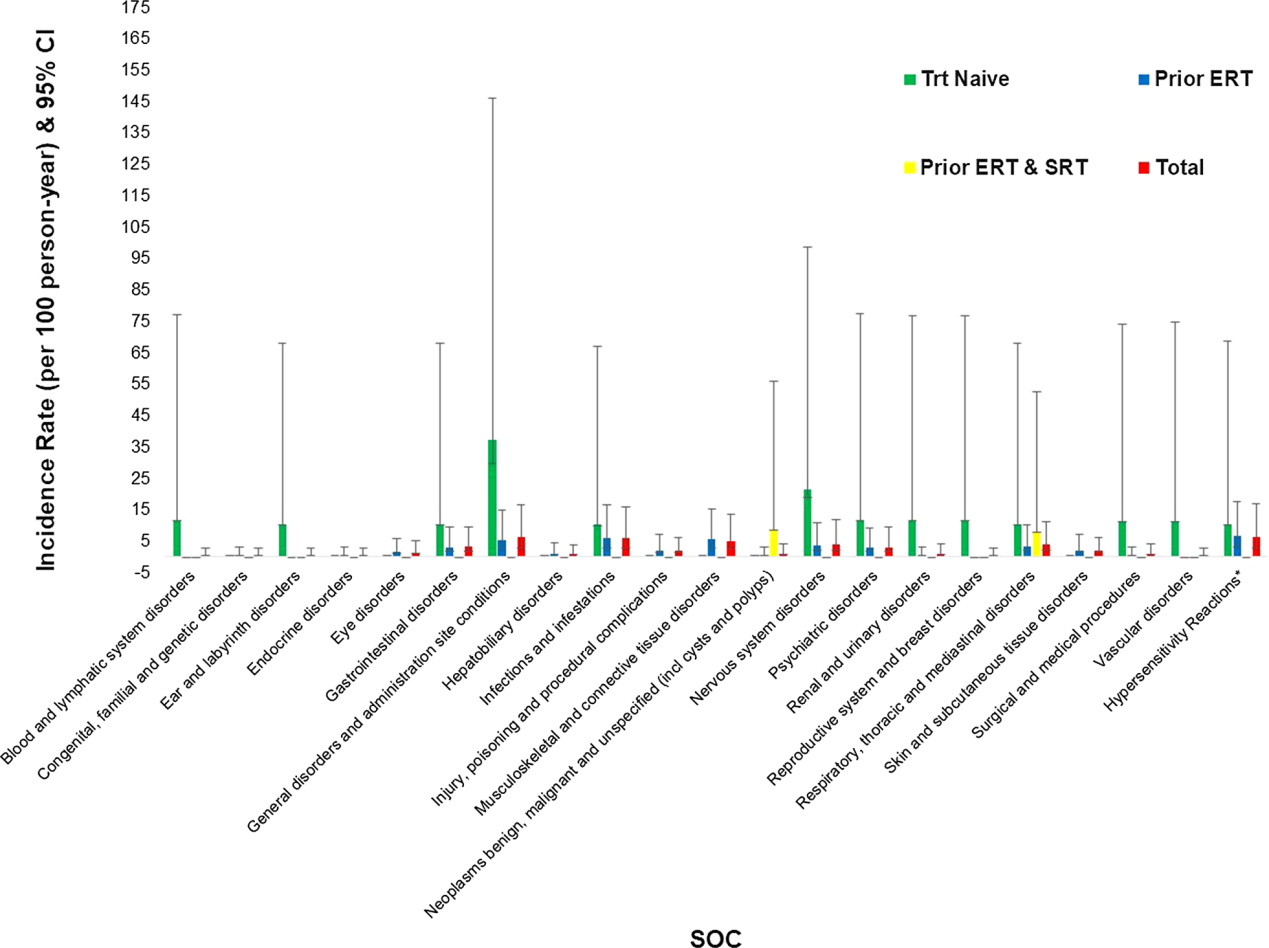
Exposure-adjusted incidence rate by system organ class and treatment group. *Hypersensitivity reactions include chest pain, urticaria, rash, localized edema, eye edema, eye pruritus, eye swelling, lip edema, throat irritation, and throat tightness. These patients were also counted in the associated system organ classes. *ERT* enzyme replacement therapy; *SOC* system organ class; *SRT* substrate reduction therapy; *Trt* treatment

Thirty-nine patients experienced 95 treatment-emergent AEs (TEAEs) (Table 2). The exposure-adjusted incidence rates per 100 person-years by SOC and treatment group are summarized in Fig. 1 and in the Additional file 1: Table. In general, the incidence rates in treatment-naïve patients were higher than those in the prior ERT group. In the treatment-naïve group, the SOC categories of blood and lymphatic system disorders, ear and labyrinth disorders, gastrointestinal disorders, general disorders and administration site conditions, hypersensitivity reactions, and infections and infestations had incidence rates per 100 person-years that were greater than 10. In contrast, in the prior ERT group, none of the SOC categories had incidence rates per 100 person-years greater than 10. In total, 12 (11.3%) patients (adults: *n* = 8; pediatric: *n* = 4) experienced 21 TEAEs that were considered related to treatment with taliglucerase alfa by the treating physician; most of these events (95.2%) occurred in the prior ERT group (Table 2).

Overall, eight patients (all adults) experienced severe AEs (Table 2); only two experienced treatment-related severe AEs (chest pain, back pain, and systemic lupus erythematosus), which were also reported as SAEs. All of these severe AEs occurred in the prior ERT group.

### Effectiveness assessment

The effectiveness of taliglucerase alfa was analyzed using data from patients with up to 2 years of follow-up because only a small proportion (6.6%) of patients had follow-up data beyond year 2. In the treatment-naïve group, only year 1 effectiveness data are presented below, as there were no patients followed 2 years as of the cutoff date for the study.

#### Hemoglobin concentration

The mean (standard deviation [SD]) baseline hemoglobin concentration in the treatment-naïve group (11.6 [1.4] g/dL) was lower compared with the prior ERT and prior ERT and SRT groups (13.3 [1.7] g/dL and 13.8 [1.5] g/dL, respectively; Table 3). In the treatment-naïve group, the mean (SD) increase in hemoglobin concentration was 1.2 (1.3) g/dL at year 1 (mean percent increase of 10.5%). The mean hemoglobin concentration in the prior ERT group remained stable at years 1 and 2.

**Table 3:** Summary of hemoglobin concentration and platelet count at baseline and follow-up, all patients

Endpoint	Visit (statistics)	Treatment-naïve group (*n* = 8)	Prior ERT group (*n* = 93)	Prior ERT and SRT group (*n* = 5)^a^	Total patients (*N* = 106)
Hemoglobin, mean (SD), g/dL	Baseline	*n* = 8	*n* = 83	*n* = 4	*n* = 95
	Absolute values	11.6 (1.4)	13.3 (1.7)	13.8 (1.5)	13.2 (1.7)
	Year 1 (month 12)	*n* = 3	*n* = 54	*n* = 1	*n* = 58
	Absolute values	13.0 (0.8)	13.1 (1.5)	14.4	13.2 (1.5)
	Change from baseline^b^	1.2 (1.3)	0 (0.8)	− 0.7	0 (0.9)
	% change from baseline^b^	10.5 (11.3)	0.1 (6.7)	− 4.6	0.5 (7.2)
	Year 2 (month 24)	*n* = 0	*n* = 44	*n* = 0	*n* = 44
	Absolute values	–	13.4 (1.3)	–	13.4 (1.3)
	Change from baseline^b^	–	0.1 (0.9)	–	0.1 (0.9)
	% change from baseline	–	0.7 (7.3)	–	0.7 (7.3)
Platelet count without splenectomy, mean (SD), 10^3^/μL	Baseline	*n* = 5	*n* = 66	*n* = 2	*n* = 73
	Absolute values	128.8 (30.3)	169.2 (77.0)	202.5 (21.9)	167.3 (74.5)
	Year 1 (month 12)	*n* = 3	*n* = 41	*n* = 1	*n* = 45
	Absolute values	137.7 (35.4)	168.0 (52.4)	187.0	166.4 (51.2)
	Change from baseline^b^	17.0 (18.2)	7.9 (42.5)	− 31.0	7.6 (41.2)
	% change from baseline^b^	14.3 (14.4)	10.7 (32.4)	− 14.2	10.4 (31.2)
	Year 2 (month 24)	*n* = 0	*n* = 36	*n* = 0	*n* = 36
	Absolute values	–	193.7 (80.0)	–	193.7 (80.0)
	Change from baseline^b^	–	22.1 (38.1)	–	22.1 (38.1)
	% change from baseline^b^	–	20.0 (35.1)	–	20.0 (35.1)
Platelet count with splenectomy, mean (SD), 10^3^/μL	Baseline	*n* = 3	*n* = 16	*n* = 0	*n* = 19
	Absolute values	183.7 (28.3)	250.0 (100.2)	–	239.7 (95.3)
	Year 1 (month 12)	*n* = 0	*n* = 9	*n* = 0	*n* = 9
	Absolute values	–	235.1 (87.8)	–	235.1 (87.8)
	Change from baseline^b^	–	− 7.0 (60.7)	–	− 7.0 (60.7)
	% change from baseline^b^	–	1.7 (25.2)	–	1.7 (25.2)
	Year 2 (month 24)	*n* = 0	*n* = 7	*n* = 0	*n* = 7
	Absolute values	–	281.1 (89.2)	–	281.1 (89.2)
	Change from baseline^b^	–	− 2.1 (68.1)	–	− 2.1 (68.1)
	% change from baseline^b^	–	1.0 (27.8)	–	1.0 (27.8)

^a^One patient was reported under the “Prior ERT and SRT” group due to erroneous recording of some of his ERT treatments as SRTs in the study database

^b^Summaries for change and percent change from baseline were calculated from the pair of observations from patients who completed both post-baseline and baseline measurement

#### Platelet count

At baseline, the mean platelet count was higher in both the prior ERT group and the prior ERT and SRT group compared with the treatment-naïve group, as expected, regardless of the splenectomy status (i.e. patients with a history of splenectomy and patients with no history of splenectomy). Furthermore, at baseline, the mean platelet count in patients with splenectomy was higher than those without splenectomy in the treatment-naïve group (183.7 · 10^3^/μL vs. 128.8 · 10^3^/μL, respectively) and the prior ERT group (250.0 · 10^3^/μL vs. 169.2 · 10^3^/μL, respectively; Table 3). At the year 1 follow-up visit, a mean increase of 17.0 · 10^3^/μL from baseline (mean percent increase of 14.3%) to near-normal levels was observed in patients without splenectomy in the treatment-naïve group. In the prior ERT group, mean platelet count increased from baseline (mean percent increase from baseline of 10.7% at year 1 and 20.0% at year 2) in patients without splenectomy; however, the value remained stable post-splenectomy during the follow-up in patients who had undergone splenectomy.

#### Liver volume

At baseline, the median liver volume by multiples of normal (MN) of 2.4 was higher in the prior ERT group compared with 1.4 in the treatment-naïve group (Table 4). In the treatment-naïve group, with only two patients followed through year 1, the percent change from baseline in liver volume by MN decreased by 5.3% in one patient and increased by 132.1% in the other patient. In the prior ERT group, a median of 1.5% increase was observed at year 1, while the median liver volume MN decreased by 3.0% at year 2.

**Table 4:** Summary of organ volume by MN at baseline and follow-up, all patients

Organ	Visit (statistics)	Treatment-naïve group (*n* = 8)	Prior ERT group (*n* = 93)	Prior ERT and SRT group (*n* = 5)^a^	Total patients (*N* = 106)
Liver, median (range)	Baseline	*n* = 6	*n* = 68	*n* = 4	*n* = 78
	Absolute values	1.4 (0.3–2.1)	2.4 (0.1–10.9)	0.7 (0.6–0.8)	2.2 (0.1–10.9)
	Year 1 (month 12)	*n* = 2^b^	*n* = 40	*n* = 0	*n* = 42
	Absolute values	0.9 (0.6, 1.2)	2.4 (0.5–6.2)	–	2.3 (0.5–6.2)
	Change from baseline^c^	0.1 (− 0.1, 0.4)	0.0 (− 5.3–1.0)	–	0.0 (− 5.3–1.0)
	% change from baseline^c^	63.4 (− 5.3, 132.1)	1.5 (− 50.9–877.5)	–	1.5 (− 50.9–877.5)
	Year 2 (month 24)	*n* = 0	*n* = 29	*n* = 0	*n* = 29
	Absolute values	–	2.7 (0.6–4.7)		2.7 (0.6–4.7)
	Change from baseline^c^	–	− 0.1 (− 6.6–2.3)	–	− 0.1 (− 6.6–2.3)
	% change from baseline^c^	–	− 3.0 (− 60.0–159.3)	–	− 3.0 (− 60.0–159.3)
Spleen, median (range)	Baseline	*n* = 4	*n* = 53	*n* = 2	*n* = 59
	Absolute values	9.5 (6.8–18.1)	4.9 (0.6–121.5)	1.8 (1.6, 2.0)	5.3 (0.6–121.5)
	Year 1 (month 12)	*n* = 2^b^	*n* = 33	*n* = 0	*n* = 35
	Absolute values	4.5 (1.9, 7.0)	4.9 (1.7–120.0)	–	4.9 (1.7–120.0)
	Change from baseline^c^	− 9.7 (− 11.1, − 8.3)	0.0 (− 82.5–42.3)	–	− 0.2 (− 82.5–42.3)
	% change from baseline^c^	− 71.1 (− 81.0, − 61.3)	− 0.9 (− 67.9–863.0)	–	− 6.5 (− 81.0–863.0)
	Year 2 (month 24)	*n* = 0	*n* = 24	*n* = 0	*n* = 24
	Absolute values	–	4.2 (0.8–23.4)		4.2 (0.8–23.4)
	Change from baseline^c^	–	− 1.0 (− 98.1–4.2)	–	− 1.0 (− 98.1–4.2)
	% change from baseline^c^	–	− 25.0 (− 80.7–114.8)	–	− 25.0 (− 80.7–114.8)

*ERT* enzyme replacement therapy; *MN,* multiples of normal; *N* number of patients in each group; *n* number of patients contributing to summary statistics for each visit; *SD* standard deviation; *SRT* substrate reduction therapy

^a^One patient was reported under the “Prior ERT and SRT” group due to erroneous recording of some of his ERT treatments as SRTs in the study database

^b^The individual values were listed for *n* = 2

^c^Summaries for change and percent change from baseline were calculated from the pair of observations from patients who completed both post-baseline and baseline measurement

#### Spleen volume

At baseline, the median spleen volume by MN was lower in the prior ERT group compared with the treatment-naïve group (4.9 vs 9.5; Table 4). Median spleen size decreased by 61.3% and 81.0% from baseline for the two patients in the treatment-naïve group who had a follow-up visit at year 1. In the prior ERT group, the median spleen volume by MN decreased by 0.9% at year 1 and 25.0% at year 2.

#### Pregnancy and lactation sub-study

Among the 57 women enrolled in the Drug Registry, 29 women (51%) were of childbearing potential, defined as post-menarcheal, ≤ 2 years post-menopause, and not surgically sterilized; two became pregnant during the study and were enrolled in the pregnancy and lactation sub-study. Both patients received treatment with taliglucerase alfa during pregnancy and lactation. The pregnancy outcome for both patients was a singleton live birth without any postpartum or other complications. No congenital anomaly or birth defects were observed in the two newborns. Breastfeeding was the sole source of nutrition for both infants. No AEs were reported for these two infants.

### Pediatric patients

Sixteen of the 106 enrolled patients were aged < 18 years (treatment-naïve group, *n* = 2; prior ERT group, *n* = 14). Thirteen of 16 pediatric patients experienced 22 AEs (treatment-naïve group, two AEs; prior ERT group, 20 AEs). No serious or severe AEs were reported.

## Discussion

The multinational Drug Registry (TALIAS) aims to characterize the long-term safety (primary objective) and effectiveness (secondary objective) of taliglucerase alfa using real-world data from routine clinical practices. The first patient was enrolled in the Drug Registry in September 2013, with the intention of the Drug Registry being operational for 10 years, with the projected last patient last visit in 2023.

The Drug Registry is set up as active surveillance with no a priori AEs of special interest selected for monitoring at the design phase. Hence, for safety assessments, all events irrespective of causality were collected in the Drug Registry. Similar to findings from other studies of patients with GD treated with taliglucerase alfa, most AEs were not serious and were transient in nature, and not related to treatment with taliglucerase alfa. In multicenter clinical trials with extended follow-up (up to 30 months), commonly reported AEs were mild to moderate in intensity (e.g. infusion-related reactions, pyrexia, arthralgia, diarrhea, headache, cough, nasopharyngitis, upper respiratory tract infection, musculoskeletal pain) [8–14]. In the current analysis, vomiting, cough, cystitis, urinary tract infection, arthralgia, psoriasiform dermatitis, and peripheral edema were reported for one patient each in the prior ERT group, with an estimated incidence rate of 0.47 per 100 person-years. Four deaths occurred during the study, but no death was attributed to taliglucerase alfa. Of the 12 patients (11.3%) who experienced SAEs, two patients (1.9%) experienced four SAEs (systemic lupus erythematosus, back pain, chest pain, dyspnoea) that were deemed treatment-related; all events resolved when taliglucerase alfa was permanently discontinued.

Similar to other studies of adult and pediatric patients with GD treated with taliglucerase alfa [8–14], the interim data analysis showed improvements in clinical parameters of hemoglobin concentration, platelet count, and spleen and liver volume in previously untreated patients as well as demonstrated general stability in patients who were switched from prior GD therapies. In the pregnancy and lactation sub-study, two patients who became pregnant during the study received treatment with taliglucerase alfa during pregnancy and lactation. Breastfeeding was the sole source of nutrition for both infants. The pregnancy outcome for both patients was live birth with no complications or AEs reported for the infants. This is consistent with prior reports of GD, ERT, and pregnancy outcomes. A recent publication describing outcomes of 453 pregnancies in 189 women with GD demonstrated similar percentages of normal outcomes in untreated and ERT-treated pregnancies (92.9% and 91.4%), indicating that continuation of ERT during pregnancy may be appropriate for patients with GD. In that publication, six pregnancies with exposure to taliglucerase alfa were reported, resulting in four normal outcomes, one spontaneous abortion, and one elective abortion [16].

This Drug Registry allows prospective monitoring and characterization of the long-term safety and effectiveness of taliglucerase alfa, the third FDA-approved ERT in the real-world setting. However, it is challenging to enroll patients because GD is a rare disease and physicians have numerous therapeutic options available for their patients. In addition, this Drug Registry was initiated after other GD registries were established—the International Collaborative Gaucher Group Registry was launched in 1991 and the Gaucher Disease Outcome Survey began in 2010. Our scope is more limited than these two registries and focused solely on patients treated with taliglucerase alfa. Investigators are not precluded from continuing to enter data on taliglucerase alfa-treated patients in either of the other registries. Nevertheless, there is competition for investigator time and effort that has impacted recruitment for our study. Our Drug Registry enrolled 106 patients by the data cutoff date for the current interim analysis; the number of patients is expected to increase over time. Additional study sites (e.g. in Turkey) are currently adding data, and as use of taliglucerase alfa increases, we anticipate further enrollments before the October 2023 closing date.

Certain limitations should be considered when interpreting the study data. As this is a single-arm Drug Registry, no active comparator group is available. Nonetheless, the active surveillance approach used in this study permits identification of safety signals for further refinement and evaluation. This is a feature that distinguishes TALIAS from the other registries that are less focused on safety outcomes. Furthermore, as with any observational study where physicians and patients are invited to participate and have an option to decline, not all taliglucerase alfa-treated patients are enrolled. Consequently, generalizability of findings needs to be considered. In this planned interim report, the effectiveness of taliglucerase alfa was assessed using data from patients with only ≤ 2 years of follow-up data, precluding any analyses of longer-term outcomes. In addition, as the numbers of patients in the treatment-naïve and prior combined ERT/SRT groups were quite small, caution should be exercised when interpreting data in these groups because of the small denominators and resulting numerically unstable estimates. However, these numbers are expected to increase significantly once all reporting has been completed at the end of the full study.

## Conclusions

The Drug Registry aims to provide meaningful information regarding the long-term safety and effectiveness of taliglucerase alfa using real-world data from routine clinical care. Findings from this interim analysis of Drug Registry data support the safety and effectiveness of taliglucerase alfa treatment for treatment-naïve patients with GD or for patients with GD who were previously treated with ERT. Taliglucerase alfa was well tolerated, with no new or emergent safety risks identified. Overall, safety findings were consistent with the known characteristics of taliglucerase alfa established in the pivotal clinical trials.

## Methods

### Drug Registry design and study population

**Fig. 2:**
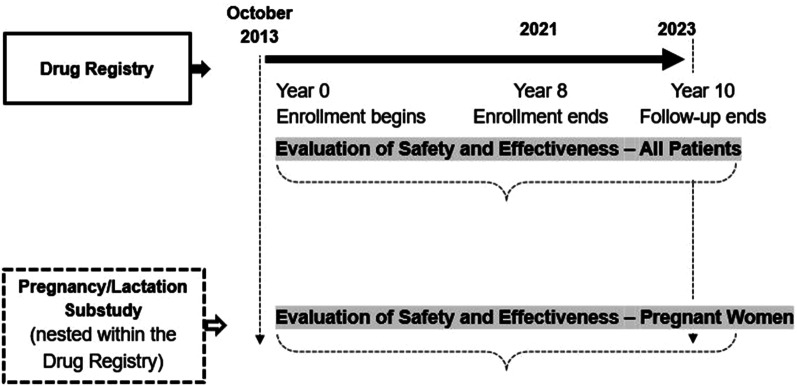
Study design

The Drug Registry is an ongoing, multinational, prospective 10-year cohort study of patients with GD in four countries (Israel, Albania, Turkey, and United States). The design of the Drug Registry and the pregnancy and lactation sub-study are shown in Fig. 2. The duration of individual patient follow-up will depend on the time of enrollment, ranging from a minimum of 2 years follow-up period to 10 years, or until the end of follow-up, whichever occurs first. The Drug Registry allowed all eligible patients with a confirmed diagnosis of GD (Types 1, 2, or 3) who are currently taking or initiating taliglucerase alfa and are willing to provide consent to be enrolled in the Drug Registry. This study is registered at the European Union electronic Register of Post-Authorisation Studies (EUPAS4721).

The Drug Registry protocol was approved by an institutional review board and/or independent ethics committee at each participating site, in accordance with local legislation and requirements.

Patient data pertaining to the safety and effectiveness of taliglucerase alfa are being obtained prospectively through routine follow-up by the treating physicians. All treatment and clinical procedures were part of the standard care for patients with GD. All patients included in the Drug Registry were expected to visit their treating physician at least annually.

Prescribing taliglucerase alfa, including treatment duration and dosage, as well as treatment switches and discontinuations, was at the discretion of the treating physicians and anticipated to correspond to the local health authority’s approved product label. Enrolled patients are being followed for the duration of participation in the Drug Registry, regardless of whether they discontinue treatment with taliglucerase alfa and/or switch treatments during the follow-up period.

### Safety endpoints

There were no prespecified safety endpoints. All causality TEAEs, treatment-related TEAEs, and SAEs occurring during follow-up were included in the safety assessment. TEAEs were defined as AEs that occur any time after the enrollment but during the reporting period in patients receiving at least one dose of taliglucerase alfa, and treatment-related TEAEs were those considered related to taliglucerase alfa by the treating physicians. SAEs were defined as any untoward medical event occurring with taliglucerase alfa treatment that was life-threatening, required inpatient hospitalization, caused prolongation of existing hospitalization, resulted in persistent or significant disability/incapacity, caused a congenital anomaly/birth defect, required intervention to prevent permanent impairment or damage, or resulted in death. All SAEs were further reconciled with Pfizer's safety database, a separate, centralized AE-monitoring database containing reports from the study investigators to the study sponsor.

### Effectiveness endpoints

Prespecified outcome measures used to evaluate the effectiveness of treatment with taliglucerase alfa included hematological assessments (i.e. hemoglobin concentration and platelet count) and organ volume (i.e. spleen and liver). Organ volume was serially measured by computed tomography, ultrasound, or magnetic resonance imaging or was estimated by palpation.

In addition to taliglucerase alfa exposure and endpoints of interests, data on patient demographics (e.g. age, sex, ethnicity, geographical location) and clinical characteristics (e.g. comorbidities, laboratory data, concomitant medications) were collected at baseline and changes were recorded during the follow-up period. For the pregnancy and lactation sub-study, pertinent data were collected from the mothers’ obstetrics/gynecology medical records and their children’s medical records.

### Data collection

All study data were entered into an electronic case report form, which was completed by authorized personnel at each site. The primary data source for the Drug Registry was the patients’ medical records.

### Study operations

The sponsor performed routine remote monitoring of data collection and management activities to ensure accuracy of the Drug Registry data and compliance with the protocol and data management plan. A Scientific Steering Committee consisting of members external to the study sponsor continually provided guidance on the study conduct, monitored progress, and advised on analyses. The committee includes experts in epidemiology, treatment of GD, and treatment of pregnant women with GD.

### Statistical analysis

The interim analysis was conducted using data from all patients who met the inclusion criteria and received at least one dose of taliglucerase alfa. Data were summarized by treatment group (i.e. treatment-naïve, prior ERT, prior ERT and SRT, and all patients) using descriptive statistics, i.e. number of patients (n), mean, standard deviation (SD), standard error, median, first and third quartile (Q1, Q3), and range for continuous variables and counts and proportions for categorical variables. Categorization of the treatment group was based on the treatment received before enrollment in the Drug Registry, including prior ERT (previously treated exclusively with ERTs), prior SRT (previously treated exclusively with SRT), prior ERT and SRT (treated with ERT and SRT), and treatment-naïve (not treated with ERT or SRT before enrollment) patients. No inferential analyses were performed, and missing values were not imputed or carried forward.

For safety assessments, the incidence rate of TEAEs was also calculated by the number of patients with events divided by the cumulative observation time (i.e. time at risk) expressed in 100 person-years.

The effectiveness of taliglucerase alfa was evaluated by treatment group using absolute value change and percent change from baseline in hemoglobin concentration (g/dL), platelet count (10^3^/μL), spleen volume (mL), and liver volume (mL); organ volumes were also expressed as MN, where applicable. The formulas for calculating organ volumes as MN are shown below:

$$\begin{aligned} {\text{Spleen volume by MN}} & = \frac{{\text{Spleen volume}}}{{{2}\,{\text{mL/kg}}*{\text{body weight}}}} \\ {\text{Liver volume by MN}} & = \frac{{\text{Liver volume}}}{{{25}\,{\text{mL/kg}}*{\text{body weight}}}} \\ \end{aligned}$$

Mean values were chosen to present hematological assessments, and median values were chosen to describe spleen and liver volumes and spleen and liver volume MN to avoid influence from outlier values.

## Supplementary Information

**Additional file 1: Table.** Number and incidence rate/100 person-years (95% CI) of all causality treatment-emergent adverse events by system organ class and treatment group.

## Data Availability

Upon request, and subject to certain criteria, conditions and exceptions (see https://www.pfizer.com/science/clinical-trials/trial-data-and-results for more information), Pfizer will provide access to individual de-identified patient data from Pfizer-sponsored global interventional clinical studies conducted for medicines, vaccines and medical devices (1) for indications that have been approved in the United States and/or European Union or (2) in programs that have been terminated (i.e. development for all indications has been discontinued). Pfizer will also consider requests for the protocol, data dictionary, and statistical analysis plan. Data may be requested from Pfizer trials 24 months after study completion. The de-identified patient data will be made available to researchers whose proposals meet the research criteria and other conditions, and for which an exception does not apply, via a secure portal. To gain access, data requestors must enter into a data access agreement with Pfizer.

## Abbreviations

AE: Adverse event
ERT: Enzyme replacement therapy
GD: Gaucher disease
MN: Multiples of normal
Q1: First quartile
Q3: Third quartile
SAE: Serious adverse event
SD: Standard deviation
SOC: System organ class
SRT: Substrate reduction therapy
TEAE: Treatment-emergent adverse event
