# Supplementary material for: Safety and effectiveness of taliglucerase alfa in patients with Gaucher disease: an interim analysis of real-world data from a multinational drug registry (TALIAS)
Source: Orphanet J Rare Dis. 2022 Apr 1;17:145. doi: 10.1186/s13023-022-02289-7 (PMC8973565; doi:10.1186/s13023-022-02289-7)
Supplement: Supplementary file 1 — Additional file 1: Table. Number and incidence rate/100 person-years (95% CI) of all causality treatment-emergent adverse events by system organ class and treatment group. [file 13023_2022_2289_MOESM1_ESM.docx]

**Supplemental Table** Number and incidence rate/100 person-years (95% CI) of all causality treatment-emergent adverse events by system organ class and treatment group

| **System organ class**  **Number of incidence rate/100 person years  (95% CI)** | **Treatment-naïve group (*n* = 8)** | **Prior ERT group (*n* = 93)** | **Prior ERT and  SRT group (*n* = 5)** | **Total patients (*N* = 106)** |
| --- | --- | --- | --- | --- |
| Blood and lymphatic system disorders | 1 | 0 | 0 | 1 |
|  | 11.73 (0.30, 65.37) | 0 (0, 0.01) | 0 (0, 0.18) | 0.42 (0.01, 2.36) |
| Congenital, familial and genetic disorders | 0 | 1 | 0 | 1 |
|  | 0 (0, 0.25) | 0.47 (0.01, 2.63) | 0 (0, 0.18) | 0.42 (0.01, 2.36) |
| Ear and labyrinth disorders | 1 | 0 | 0 | 1 |
|  | 10.36 (0.26, 57.70) | 0 (0, 0.01) | 0 (0, 0.18) | 0.42 (0.01, 2.35) |
| Endocrine disorders | 0 | 1 | 0 | 1 |
|  | 0 (0, 0.25) | 0.47 (0.01, 2.63) | 0 (0, 0.18) | 0.42 (0.01, 2.36) |
| Eye disorders | 0 | 3 | 0 | 3 |
|  | 0 (0, 0.25) | 1.45 (0.30, 4.24) | 0 (0, 0.18) | 1.30 (0.27, 3.80) |
| Gastrointestinal disorders | 1 | 6 | 0 | 7 |
|  | 10.36 (0.26, 57.70) | 2.95 (1.08, 6.42) | 0 (0, 0.18) | 3.09 (1.24, 6.36) |
| General disorders and administration site conditions | 3 | 11 | 0 | 14 |
|  | 37.25 (7.68, 108.85) | 5.33 (2.66, 9.54) | 0 (0, 0.18) | 6.13 (3.35, 10.29) |
| Hepatobiliary disorders | 0 | 2 | 0 | 2 |
|  | 0 (0, 0.25) | 0.94 (0.11, 3.41) | 0 (0, 0.18) | 0.85 (0.10, 3.06) |
| Hypersensitivity reactions^a^ | 1 | 13 | 0 | 14 |
|  | 10.44 (0.26, 58.19) | 6.51 (3.46, 11.13) | 0 (0, 0.18) | 6.27 (3.43, 10.53) |
| Infections and infestations | 1 | 12 | 0 | 13 |
|  | 10.21 (0.26, 56.86) | 5.98 (3.09, 10.45) | 0 (0, 0.18) | 5.79 (3.08, 9.91) |
| Injury, poisoning and procedural complications | 0 | 4 | 0 | 4 |
|  | 0 (0, 0.25) | 1.95 (0.53, 4.99) | 0 (0, 0.18) | 1.75 (0.48, 4.47) |
| Musculoskeletal and connective tissue disorders | 0 | 11 | 0 | 11 |
|  | 0 (0, 0.25) | 5.44 (2.71, 9.73) | 0 (0, 0.18) | 4.86 (2.43, 8.70) |
| Neoplasms benign, malignant and unspecified (including cysts and polyps) | 0 | 1 | 1 | 2 |
|  | 0 (0, 0.25) | 0.47 (0.01, 2.61) | 8.51 (0.22, 47.44) | 0.85 (0.10, 3.07) |
| Nervous system disorders | 2 | 7 | 0 | 9 |
|  | 21.41 (2.59, 77.34) | 3.49 (1.41, 7.20) | 0 (0, 0.18) | 4.03 (1.84, 7.65) |
| Psychiatric disorders | 1 | 6 | 0 | 7 |
|  | 11.77 (0.30, 65.56) | 2.90 (1.07, 6.32) | 0 (0, 0.18) | 3.06 (1.23, 6.30) |
| Renal and urinary disorders | 1 | 1 | 0 | 2 |
|  | 11.69 (0.30, 65.14) | 0.48 (0.01, 2.66) | 0 (0, 0.18) | 0.86 (0.10, 3.12) |
| Reproductive system and breast disorders | 1 | 0 | 0 | 1 |
|  | 11.67 (0.30, 65.02) | 0 (0, 0.01) | 0 (0, 0.18) | 0.42 (0.01, 2.36) |
| Respiratory, thoracic and mediastinal disorders | 1 | 7 | 1 | 9 |
|  | 10.37 (0.26, 57.76) | 3.33 (1.34, 6.85) | 7.97 (0.20, 44.38) | 3.87 (1.77, 7.34) |
| Skin and subcutaneous tissue disorders | 0 | 4 | 0 | 4 |
|  | 0 (0, 0.25) | 1.96 (0.53, 5.01) | 0 (0, 0.18) | 1.75 (0.48, 4.49) |
| Surgical and medical procedures | 1 | 1 | 0 | 2 |
|  | 11.26 (0.28, 62.75) | 0.47 (0.01, 2.63) | 0 (0, 0.18) | 0.85 (0.10, 3.08) |
| Vascular disorders | 1 | 0 | 0 | 1 |
|  | 11.39 (0.29, 63.46) | 0 (0, 0.01) | 0 (0, 0.18) | 0.42 (0.01, 2.36) |

^a^Hypersensitivity reactions includes the preferred terms: chest pain, urticaria, rash, localised oedema, eye oedema, eye pruritus, eye swelling, lip oedema, throat irritation, throat tightness. These preferred terms have been counted in their associated system organ classes as well.
